# A microbial consortium constructed with gut microbes of Chinese native chicken breeds protects chicks against *Salmonella* infection

**DOI:** 10.1186/s40104-026-01447-2

**Published:** 2026-06-26

**Authors:** Yimei Feng, Meihong Zhang, Fengwenhui Zhang, Guanyu Hou, Jinxin Zhang, Shuran Zhao, Yuqing Feng, Dan Liu, Dahai Xu, Yongfei Hu

**Affiliations:** 1 Sanya Institute, China Agricultural University, Sanya, 572025 Hainan China; 2 State Key Laboratory of Animal Nutrition and Feeding, College of Animal Science and Technology, China Agricultural University, Beijing, 100193 China; 3 Tropical Crops Genetic Resources Institute, Chinese Academy of Tropical Agricultural Sciences, Haikou, 571101 Hainan China

**Keywords:** Chick, Gut microbiota, Microbial consortium, *Salmonella* Typhimurium

## Abstract

**Background:**

The gut microbiota plays a crucial role in protecting chickens against colonization by enteric pathogens, such as *Salmonella*. Native chickens, including Wenchang chicken and Danzhou chicken, harbor a highly diverse gut microbiota, which may contain anti-pathogenic strains. This study aimed to construct a microbial consortium from these native chicken breeds and evaluate its efficacy in protecting chicks from *Salmonella* infection.

**Results:**

A collection of 273 bacterial strains, representing 61 species, was isolated from the cecal digesta of Wenchang chicken and Danzhou chicken. From this collection, 29 strains were identified as exhibiting in vitro inhibitory activity against the indicator pathogens. Genomic analysis of the constituent strains in consortium BL6 revealed the genetic potential for producing antimicrobial peptides and secondary metabolites, suggesting a mechanistic basis for the inhibition. In a chick model challenged with *S*. Typhimurium, BL6 mitigated intestinal damage by promoting crypt restoration, enhanced barrier function, and enriched *Lactobacillus*, collectively contributing to improved intestinal health.

**Conclusions:**

These findings underscore the potential of a microbial consortium derived from the gut microbes of native chickens in combating *Salmonella* infection. This research offers valuable insights for developing innovative strategies to prevent and treat pathogen infections in broilers.

**Supplementary Information:**

The online version contains supplementary material available at 10.1186/s40104-026-01447-2.

## Background

Poultry has emerged as one of the most widely consumed meats globally, a trend that has fueled the rapid growth of the poultry industry over the past few decades [1]. The dense and complex gut microbiota in chickens performs a wide range of critical functions, including nutrient digestion [2], immune system development [3], and growth performance enhancement [4]. These roles highlight the importance of gut microbiota for chicken health and productivity. In recent years, manipulating gut microbiota has increasingly been recognized as a feasible strategy to inhibit pathogenic infections [5]. For example, via fecal microbiota transplantation (FMT), a success rate of over 90% has been achieved in the treatment of recurrent *Clostridioides difficile* infection [6]. This microbiota-based intervention can be traced back to earlier findings in poultry experiment. Long before FMT gained widespread attention in human medicine, Nurmi and Rantala [7] observed that inoculating chicks with feces from healthy adult chickens could effectively prevent *Salmonella* infection. Recently, attempts have been made to identify, investigate and validate the core species in the microbiome-mediated pathogenic infections, and a strategy of using individual species or their consortia for the prevention of pathogenic infection is defined as precision microbiome reconstruction [8].

In poultry industry, *Salmonella* infection stands as a persistent challenge. When chickens are infected with *Salmonella*, the bacteria can contaminate the meat during the slaughter and processing stages. In the case of eggs, *Salmonella* can infect the ovaries of hens, leading to the presence of the bacteria inside the eggs even before they are laid. The consumption of *Salmonella*-contaminated meat and eggs is one of the primary causes of human salmonellosis [9]. Eliminating *Salmonella* colonization in poultry remains challenging due to its transmission characteristics. Specifically, it is vertically transmitted to chicks through eggs or spreads rapidly within flocks via fecal-oral route in high-density farming environments. Antibiotics remain a widely used control strategy. However, *Salmonella* evades host surveillance and defense via multiple mechanisms to invade and proliferate, making antibiotics ineffective for complete eradication, and the extensive use of antibiotics has made antibiotic resistance a global public health concern [10, 11]. Consequently, efforts have been made to reduce the infection of *Salmonella* as well as other pathogens through gut microbiota-based intervention approaches. This has been achieved by inoculating chickens with complex mixtures of synthetic microbial consortia, such as continuous-flow culture isolated from the cecal digesta of adult broiler chickens [12], 76 different bacterial isolates originating from the chicken cecum [13], and culture of competitive exclusion microbiota colonizing on the mucosal surface of the cecum [14]. However, due to the complexity of these mixtures, accurately delineating how they function and improving their efficacy are still difficult tasks, and there is a continuous need to develop targeted anti-pathogen microbial consortia to combat infections.

Studies have indicated that the gut microbiota of local chicken breeds differs from that of industrial broilers in terms of microbial species composition and functional profiles [15]. Evidence has suggested that such differences play a crucial role in conferring stronger *Salmonella* colonization resistance on local chicken breeds [16]. Wenchang chicken and Danzhou chicken are excellent local chicken breeds in Hainan Province, China. The official release of the Chinese *National breed list of livestock and poultry genetic resources (2024 edition)* [17] highlights their significance as genetic asset with substantial market value and application potential. With a long history of being raised in the region, these breeds are deeply ingrained in the local farming culture and have evolved unique genetic characteristics and strong resistance to pathogen infection, making them well-adapted to the tropical and subtropical climates of Hainan. However, research on the gut microbiota of Wenchang chicken and Danzhou chicken remains notably limited. Whether the gut microbiota of these chicken breeds contributes to their outstanding *Salmonella* infection resistance remains unclear.

In this study, we characterized the gut microbial composition of these two chicken breeds and established a bacterial culture collection from their gut microbiota. We further constructed a defined microbial consortium (BL6) using the *Salmonella*-inhibitory strains screened from the culture collection. BL6 was then evaluated for its *Salmonella*-eliminating capacity using an early intervention trial with 1-day-old chick model. Our findings demonstrate the efficacy of the designed BL6 in combating *Salmonella* infection in chicks, and present a potential strategy for developing defined communities as anti-*Salmonella* therapeutics in the poultry industry.

## Methods

### Sample collection

Intestinal digesta samples were obtained from 160-day-old healthy hens (Wenchang chickens and Danzhou chickens, *n* = 10 per breed) at local conservation farms in Hainan Province, China. Chickens with consistent plumage and body weight were selected and euthanized by electrical stunning for collection of mid-ileal and mid-cecal digesta.

### DNA extraction and 16S rRNA gene amplicon sequencing

Genomic DNA was extracted from intestinal digesta using the QIAamp Fast DNA Stool Mini Kit (Qiagen, Valencia, CA, USA). Both second-generation and third-generation sequencing were utilized. Specifically, for the analysis of the ileal microbiome in Arbor Acres broiler chicks, the V3–V4 region of the bacterial 16S rRNA gene was amplified using primers 341F (5′-CCTACGGGNBGCASCAG-3′)/805R (5′-GACTACNVGGGTATCTAATCC-3′) [18] and sequenced on Illumina HiSeq 2500 platform (Institute of Microbiology, Chinese Academy of Sciences). Separately, for the analysis of the cecal and ileal microbiome of local chickens, the full-length 16S rRNA gene was amplified with barcoded primers 27F (5′-AGRGTTTGATYNTGGCTCAG-3′)/1492R (5′-TASGGHTACCTTGTTASGACTT-3′) [19] and sequenced on PacBio Sequel II system at Biomarker Technologies Co., Ltd.

### Sequencing data processing and bioinformatics analysis

Raw sequencing data from both platforms were processed through an identical QIIME2 pipeline (v2023.9) [20]. Briefly, DADA2 (v1.10.0) [21] was used for denoising and ASV inference for both platforms, with an additional read assembly step for Illumina paired-end data prior to ASV inference. Sequences were further filtered to remove contaminants, rare, and unclassified ASVs. Taxonomy was assigned using a naive Bayes classifier trained on the SILVA database (v138) [22]. For alpha and beta diversity analysis, Chao1, Shannon indices and Bray–Curtis-based PCoA were calculated in MicrobiomeAnalyst (https://www.microbiomeanalyst.ca/) (accessed on 5 December 2023). Bacterial discriminative features were identified with LEfSe (http://huttenhower.sph.harvard.edu/galaxy/) (accessed on 1 December 2023).

### Strain isolation

Bacterial isolation followed previous study with modifications [23]. Briefly, cecal digesta was diluted and plated on the media (Table S1), then incubated in an anaerobic environment within a MUNRO AW 400SG anaerobic workstation (maintaining an atmosphere of 10% H_2_, 10% CO_2_, and 80% N_2_; Munro, UK) at 37 °C for several days. Selected colonies were identified by amplifying the 16S rRNA gene with primers 27F (5′-AGAGTGTGATCCTGGCTCAG-3′) and 1492R (5′-TACGGTTACCTTGTTACGACTT-3′). Isolates with > 97.82% [16] sequence identity to validated species in EZBioCloud (https://www.ezbiocloud.net) (accessed on 10 March 2023) [24] were designated as known. Those ≤ 97.82% were further analyzed using the NCBI 16S ribosomal RNA sequence database and considered novel taxa candidates. All strains were stored at −80 °C in 20% glycerol.

### Screening for inhibition activity of pathogens

The inhibitory effects of all strains on *Salmonella* Typhimurium, *Clostridium perfringens*, and *Escherichia coli* were evaluated by the bilayer agar plate assay described by Wang et al. [25]. Bacterial cell-free supernatants (CFS) were prepared by centrifugation and filtration. The pathogenic bacteria were inoculated into liquid LB agar at a concentration of 1% and then poured into the sterile Petri dishes. After the agar solidified, 30 μL of CFS was dispensed into Oxford cup on the plates. Culture medium and ampicillin served as negative and positive controls, respectively. The plates were incubated at 37 °C for 24 h, and inhibition zones were measured.

### Whole-genome sequencing

Genomic DNA of the BL6 strains was extracted using the DNeasy Blood and Tissue Kit (Qiagen, Hilden, Germany). Sequencing was performed on the Illumina HiSeq 2500 platform at Shanghai Majorbio Bio-pharm Technology Co., Ltd. Raw reads were quality-trimmed with Trimmomatic (v0.32) [26] and de novo assembled using IDBA-UD (v1.1.1) [27]. The assembled genomes were annotated with Prokka (v1.14.6) [28] for CDS, rRNA, tRNA, and other features. Functional annotations were derived from the KEGG [29] and COG [30] databases, and secondary metabolite gene clusters were predicted with antiSMASH (v6.0.1) [31].

### Preparation of synthetic microbial consortium

To integrate the BL6 strains into a simplified synthetic consortium, we adapted a standardized cultivation protocol based on our previous work [32]. Briefly, each strain in BL6 was subcultured daily by transferring a 1:10 inoculum into fresh growth medium. After 72 h of cultivation, each bacterial culture was concentrated and normalized to an OD_600_ of 1.3, which corresponds to approximately 10^9^ CFU/mL for *Escherichia coli* according to prior studies [33]. Subsequently, the cultures were combined in equal proportions, centrifuged, washed, and finally resuspended in PBS with 20% glycerol to a 10-fold volume.

### Animals, experimental design and* Salmonella* Typhimurium challenge

This experiment was conducted in accordance with the Chinese guidelines for animal welfare. Ninety 1-day-old male Arbor Acres chicks hatched on the same day were procured from Beijing Dafa Chia Tai Co., Ltd. and housed in a poultry farm (Zhuozhou, Hebei, China). Chicks were randomly assigned to three weight-matched groups (*n* = 30/group, 6 replicates of 5 chicks): CON (sterile PBS), PC (*S*. Typhimurium challenge), and BL6 (BL6 intervention + *S*. Typhimurium challenge). From d 1 to d 4, the BL6 group received oral gavage of BL6 (1 × 10^8^ CFU/d), while CON and PC groups received PBS. On d 5–6, both PC and BL6 groups were challenged with *S*. Typhimurium (1 × 10^8^ CFU/d), which was quantified using the same OD_600_ normalization method as described for the BL6. CON group received PBS. All chicks were raised under controlled conditions with ad libitum access to antibiotic-free feed and water. Room temperature, humidity, ventilation, and lighting were automatically controlled following the Arbor Acres broiler management guidelines. The feed was prepared in accordance with the Chinese Chicken Feeding Standard (NY/T-33-2004) [34], with composition and nutrient levels detailed in Table S8. At 7 and 14 days of age, two chicks per replicate were humanely euthanized by electrical stunning for sample collection. Serum, mid-ileal digesta and tissues were collected and processed for downstream analysis.

### Histopathology

Ileum samples fixed in 4% paraformaldehyde solution were processed through dehydration, paraffin embedding, sectioning, and hematoxylin–eosin (HE) staining at Wuhan Servicebio Technology Co., Ltd. Stained sections were imaged using a microscope (Leica DM750). Villus height (VH) and crypt depth (CD) were measured, and VH:CD ratio was calculated.

### Serum biochemistry analysis

Serum ALT and AST levels were measured using an automatic biochemical analyzer (Boke BK-200VET, Shandong, China). Immunoglobulins (IgA, IgM, and IgG) were quantified via two-site sandwich enzyme immunoassay using commercial kits (Tiangen Biotechnology Co., Ltd.).

### Total RNA isolation and RT-qPCR

Total RNA was extracted from intestinal tissue using TRIzol reagent (Genestar). After assessing purity and concentration by NanoDrop Micro UV–Vis Spectrophotometer (Thermo Scientific, MA, USA), cDNA was synthesized with the Primer Script RT Reagent kit (Beyotime Biotechnology). RT-qPCR was performed on a QuantStudio 3 system (Applied Biosystems, CA, USA) using SYBR Premix (Applied Biosystems), with gene-specific primers (Sangon Biotech, Table S9). The thermal profile was: 95 °C for 2 min; 40 cycles of 95 °C for 15 s and 60 °C for 20 s; melting curve stage at 95 °C for 15 s, 60 °C for 15 s and 95 °C for 15 s. Melt curve analysis confirmed specificity. Relative expression was calculated by the 2^−ΔΔCt^ method using β-actin as the reference gene.

### Statistical analysis

Statistical analysis was performed using SPSS (v27.0) for the *Salmonella* challenge animal experiment. Homogeneity of variance was examined by Levene’s test. Data with homogeneous variance were analyzed by one-way ANOVA with LSD post-hoc test, whereas data with heterogeneous variance were analyzed by Welch’s ANOVA with Tamhane’s T2 post-hoc test. Data are presented as mean ± SEM. Each treatment included three independent replicates.

For microbiota analysis, alpha diversity was assessed by the Wilcoxon test, beta diversity by PERMANOVA, and differentially abundant taxa were identified using LEfSe, which uses the nonparametric Kruskal–Wallis rank-sum test combined with LDA to evaluate feature effect size (LDA score > 2). Statistical significance was set at *P* < 0.05, *P* < 0.01 and *P* < 0.001.

## Results

### Characterization of the gut microbiota in Wenchang chicken and Danzhou chicken

To characterize the microbial community structure in the gut microbiota of Wenchang chicken and Danzhou chicken, full-length 16S rRNA genes from both ileal and cecal samples of these two breeds were sequenced using a third-generation sequencer with the Circular Consensus Sequencing (CCS) approach. A total of 517,620 raw CCS reads were obtained from 40 collected samples (including 10 samples each from the cecum and ileum of Wenchang chickens and Danzhou chickens). Each sample generated at least 9,160 clean CCS reads, with an average of 12,941 clean CCS reads per sample. After quality control filtering, forward reads with an average length of approximately 10,098 bp were retained, and 8,324 ASVs were identified.

**Fig. 1:**
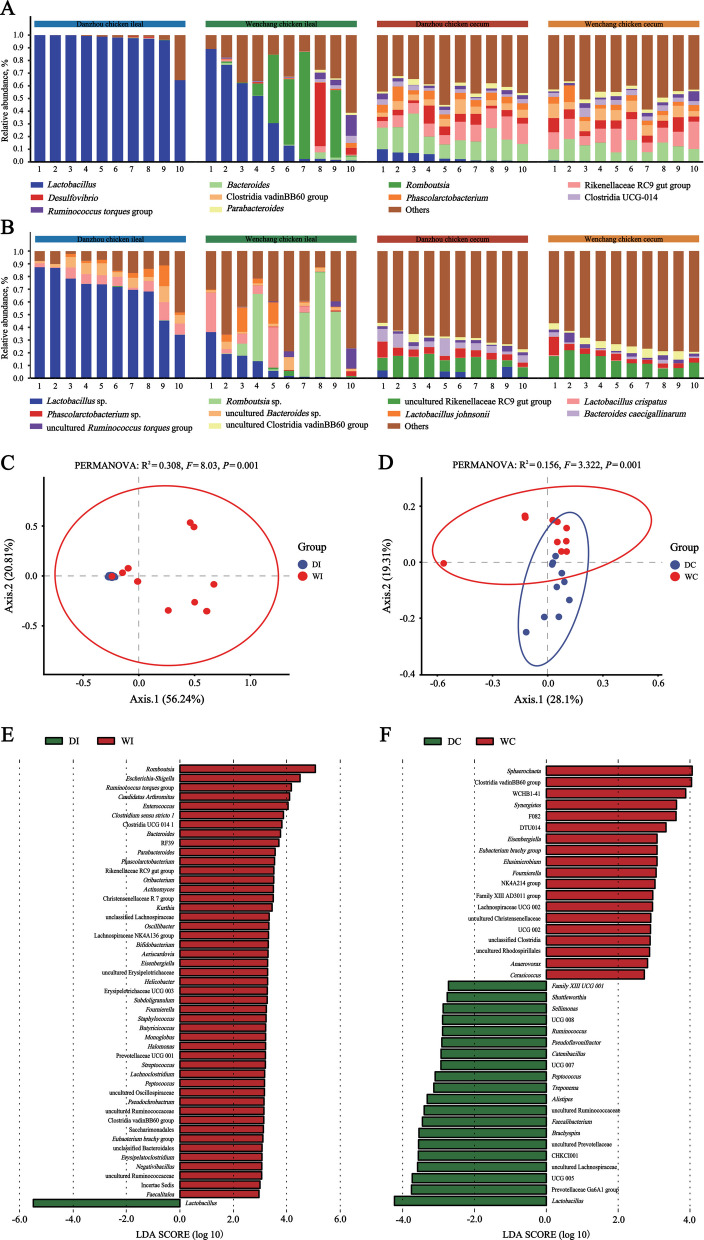
Gut microbiota composition of Wenchang chicken and Danzhou chicken. **A** and **B** Composition of bacterial communities at the genus (**A**) and species (**B**) levels, present in ileal and cecal digesta of Wenchang chicken and Danzhou chicken. **C** and** D** PCoA of bacterial communities in the ileal (**C**) and cecal (**D**) digesta of Wenchang chicken and Danzhou chicken. **E** and **F** LEfSe analysis identifying differentially abundant bacterial taxa in the ileal (**E**) and cecal (**F**) digesta of Wenchang chicken and Danzhou chicken

After eliminating redundancy from the original sequencing data, a total of 23 phyla and 312 genera were annotated using the SILVA database. At the phylum level, Firmicutes was the dominant phylum in the ileal microbiota, though its abundance varied significantly between the two breeds. Specifically, in Danzhou chicken, Firmicutes accounted for 99.06%, as opposed to 83.69% in Wenchang chicken (Fig. S1A and B). The cecal microbiota of the two breeds showed similar relative abundance patterns, with Firmicutes and Bacteroidetes as the co-dominant phyla, followed by Desulfobacterota and Verrucomicrobiota (Fig. S1C and D). At the genus level, *Lactobacillus* (94.73%) was absolutely dominant in the ileum of Danzhou chicken, with extremely low relative abundances of other microbial taxa (Fig. 1A). In contrast, the ileal microbial community of Wenchang chicken was co-dominated by *Lactobacillus* (34.04%) and *Romboutsia* (30.80%) (Fig. 1A). The cecal microbiota of both breeds shared a similar compositional profile. The community was predominantly composed of the genera *Bacteroides*, *Desulfovibrio*, and *Phascolarctobacterium*, alongside the Rikenellaceae RC9 gut group and Clostridia vadinBB60 group (Fig. 1A). Notably, a large number of uncultured species were detected in the gut of both breeds (Fig. 1B). Specifically, in the cecum, the relative abundances of uncultured species reached 63.47% in Wenchang chicken and 56.50% in Danzhou chicken, respectively. This finding suggests a high level of uncharacterized microbial diversity in these local breeds.

To further investigate differences in gut microbial community structures between Wenchang chicken and Danzhou chicken, PCoA was performed based on Bray–Curtis dissimilarity. The PCoA plot revealed distinct clusters corresponding to the gut microbial communities of the two chicken breeds. Specifically, the ileal microbiota of the two breeds exhibited divergent clustering patterns (Fig. 1C). Danzhou chicken exhibited tighter individual distances and formed tighter clusters, indicating more stable ileal microbial communities. However, Wenchang chicken displayed a higher degree of dispersion. In contrast, the cecal microbiota structures of the two breeds exhibited more pronounced separation, with only a low level of similarity observed (Fig. 1D). LEfSe analysis further supported the presence of distinct microorganisms between the two breeds (Fig. 1E and F).

### Culturomics and identification of gut bacteria in Wenchang and Danzhou chickens

**Fig. 2:**
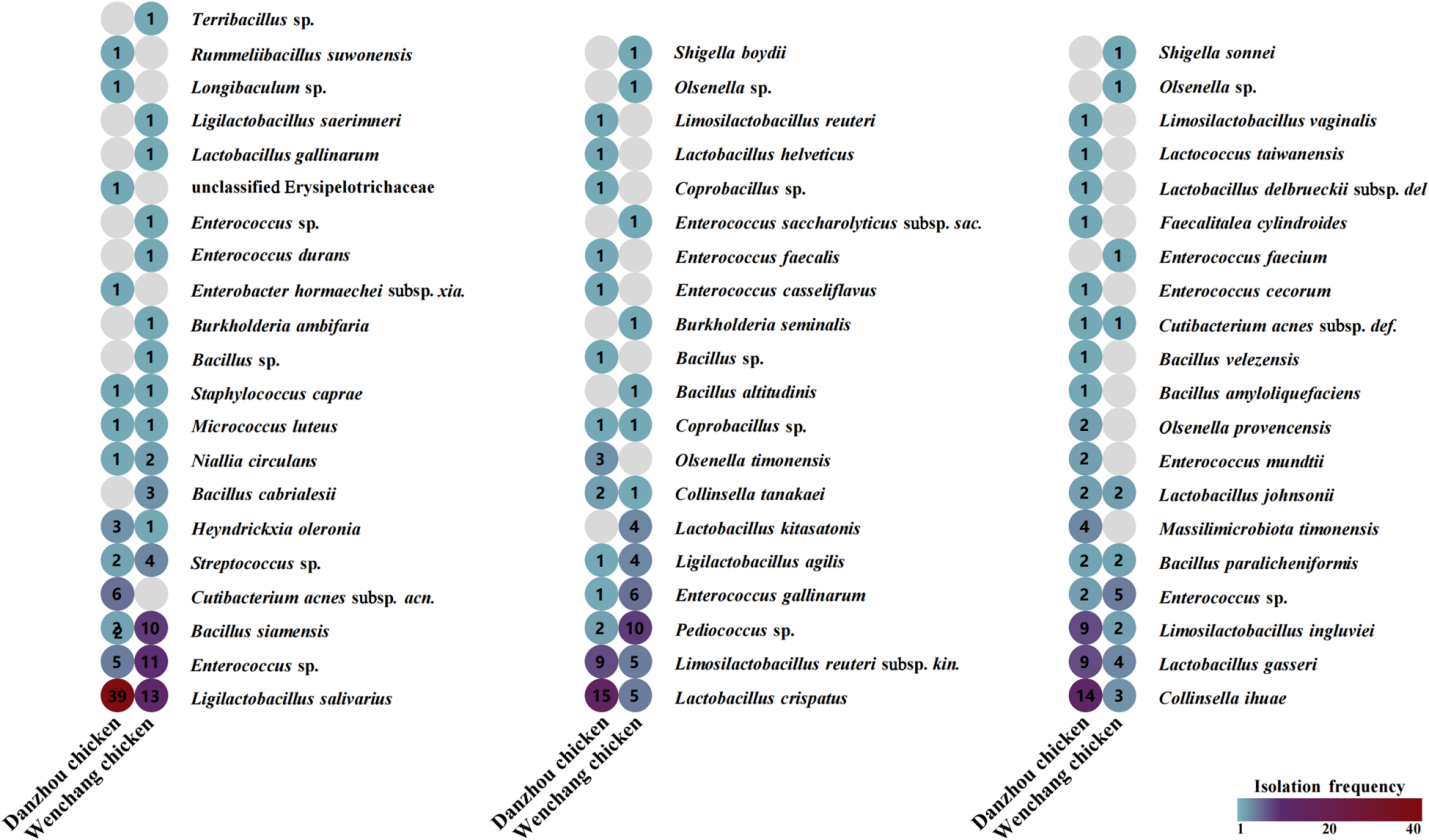
Diversity and frequency of bacterial species isolated from gut microbiota of Wenchang chicken and Danzhou chicken. The abundance and diversity of 61 bacterial species (273 strains) were counted. The numbers in each circle represent the isolation frequency of the species

Given that native chicken breeds generally exhibit enhanced pathogen resistance and adaptability [35], we hypothesized that this could be partially attributed to the unique microbiota in these chickens. We therefore isolated bacteria from both chicken breeds using differential media including MRS, LB, and GAM under anaerobic conditions. A total of 273 isolates were selected from all culture media and identified taxonomically based on 16S rRNA gene sequencing (Table S2). Figure 2 shows an overview of the frequency and diversity of the isolated species. In total, 61 distinct species were identified, with 44 species originating from the gut of Wenchang chicken and 39 species from that of Danzhou chicken. The identified species were predominantly composed of Firmicutes (47 species), followed in sequence by Actinobacteria (9 species) and Proteobacteria (5 species). Specifically, *Ligilactobacillus* (21.25%), *Lactobacillus* (15.75%), and *Enterococcus* (14.29%) accounted for 51.29% of the total number of all genera. In addition, the 16S rRNA genes of six strains were matched with “uncultured bacterium”, which may represent new bacterial species (Table S3).

### Identification of isolates conferring resistance to gut pathogens

**Fig. 3:**
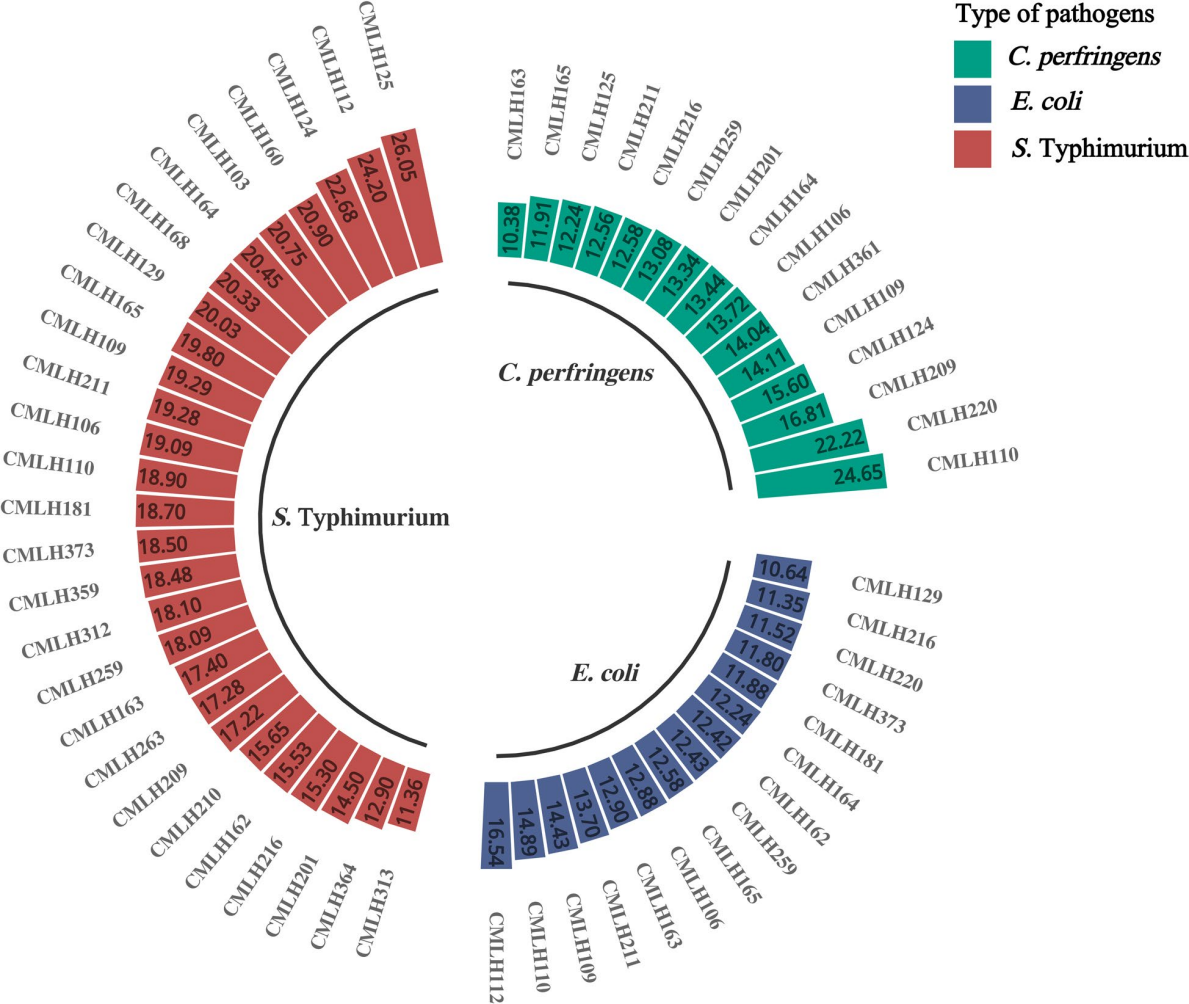
The antibacterial profile of the isolates. Using a bilayer agar assay, CFS of 273 isolates were screened against three indicator pathogens. Twenty-nine strains showed inhibitory activity against all three

To identify the isolates with the potential to inhibit common gut bacterial pathogens, we collected the culture supernatants of the 273 strains and assayed their antagonistic activities against three indicator pathogens, namely *Salmonella* Typhimurium, *Escherichia coli*, and *Clostridium perfringens*. A total of 29 strains showed varying degrees of inhibition against the three pathogens (Fig. 3 and Table S4). Given the critical public health threat of *Salmonella*, we then focused on strains with high antimicrobial activity against *S.* Typhimurium. Six strains were then selected for further analyses, including *Bacillus velezensis* CMLH125, *Ligilactobacillus salivarius* CMLH112, *Bacillus* sp. CMLH124, *Lactobacillus johnsonii* CMLH106, *Lactobacillus crispatus* CMLH109, and *Lactobacillus gallinarum* CMLH110.

### Whole-genome analysis of the anti-*Salmonella* potential of the six strains

To explore the genetic potential of the six strains in inhibiting *S.* Typhimurium, their whole-genome sequences were analyzed. Average nucleotide identity (ANI) analysis, with a cutoff of 95% for delineating bacterial species [36], was employed to confirm the taxonomic classification of the six strains. CMLH124 showed only 93.27% ANI with its closest relative, *Bacillus vallismortis*, which is below the 95% species threshold, indicating it represents a novel *Bacillus* species. The genome sizes of the six strains ranged from 1.76 to 4.13 Mb (Table 1) and their genomic structure and characteristics were visualized as a circular plot (Fig. S2). Functional annotation using the KEGG and COG databases revealed that these strains were involved in carbohydrate metabolism, amino acid metabolism, and transcription processes (Fig. S3 and S4). Further analysis highlighted the functional divergence: strains of LAB (CMLH106, CMLH109, CMLH110, CMLH112) exhibited enhanced direct involvement in metabolic pathways of carbohydrates and amino acids, while *Bacillus* strains (CMLH124, CMLH125) were proficient in metabolic regulation and material transport. Despite this divergence, both types of strains prioritized carbohydrate and amino acid metabolism as the core functional axes. Furthermore, annotation against CAZyme databases revealed that CMLH124 and CMLH125 harbored more CAZyme family genes than the other four strains (Table S5). Notably, *Bacillus* strains possessed exceptionally high gene counts across all families, especially GH and GT. Collectively, these two families accounted for more than 67.23% of the total. Using the DBAASP database, we predicted 5, 14, 5, 8, 16, and 20 antibacterial peptide-encoding genes in CMLH106, CMLH109, CMLH110, CMLH112, CMLH124, and CMLH125, respectively, all of which showed a predicted inhibitory activity against *S*. Typhimurium of more than 80% (Table S6). Additionally, we identified secondary metabolite gene clusters specifically in CMLH124 and CMLH125, including those encoding bacillibactin and pulcherriminic acid, which are compounds associated with iron chelation (Table S7).

**Table 1:** Genome characteristics of the six selected anti-*Salmonella* strains

Strain ID	Species	Genome size, Mb	GC content, %	Number of coding genes	ANI, %
CMLH106	*Lactobacillus johnsonii*	1.76	35	1,728	96.11
CMLH109	*Lactobacillus crispatus*	2.01	37	1,988	97.91
CMLH110	*Lactobacillus gallinarum*	1.97	36	1,882	98.05
CMLH112	*Ligilactobacillus salivarius*	1.84	33	1,758	97.32
CMLH124	*Bacillus* sp.	4.13	44	4,030	93.27
CMLH125	*Bacillus velezensis*	4.12	46	4,053	97.53

Genomic characteristics of the six strains with anti-*Salmonella* activity. Values were calculated using the type strain genome of each species as a reference

### BL6 mitigates *Salmonella*-induced intestinal injury through facilitation of crypt restoration

**Fig. 4:**
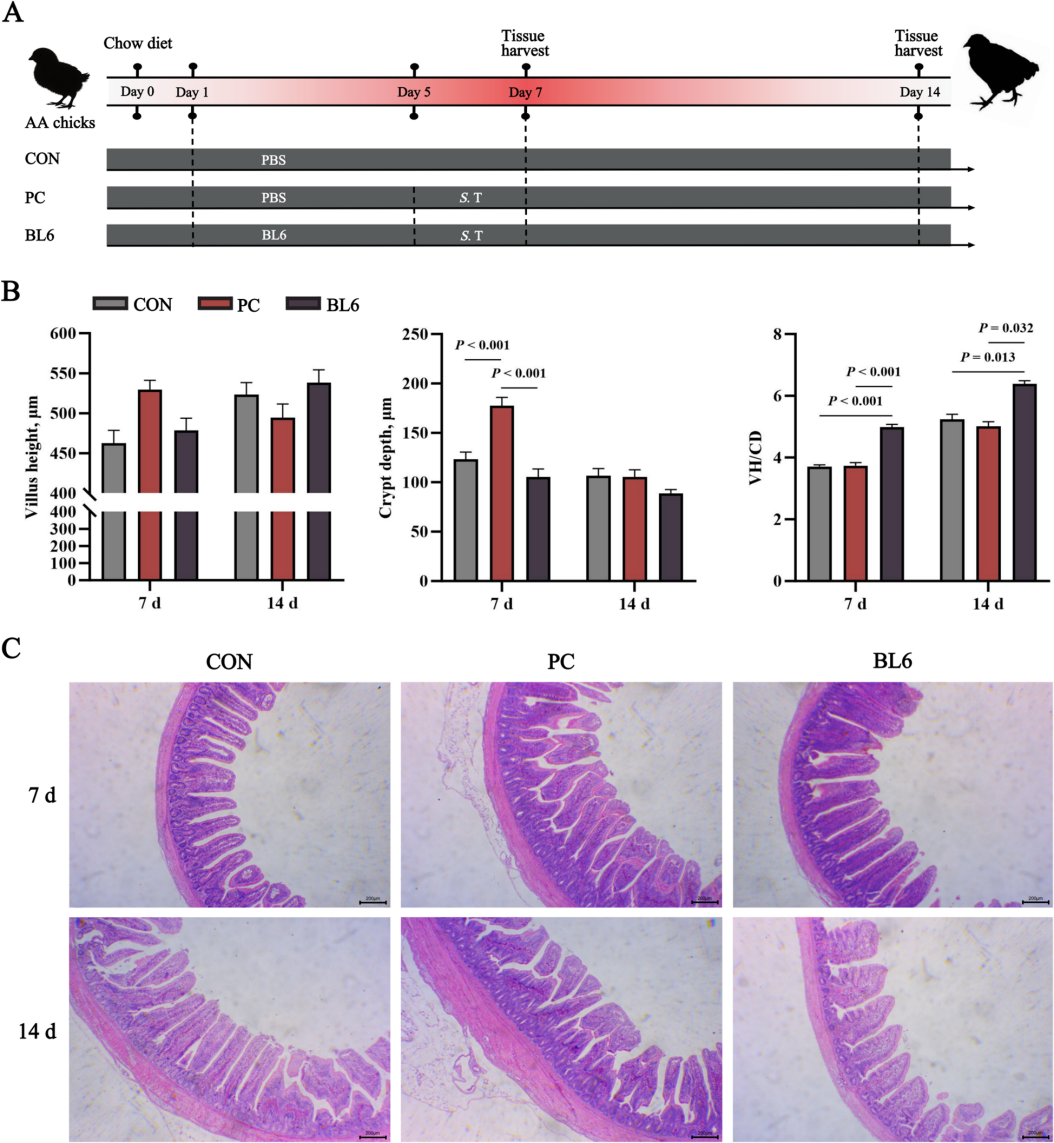
Effects of early BL6 intervention on intestinal morphology in *S*. Typhimurium challenged chicks. **A** Chick experimental design for early BL6 intervention against *S*. Typhimurium in vivo. **B** Villus height, crypt depth and villus height/crypt depth was calculated. **C** Representative Hematoxylin–eosin-stained photomicrographs of ileal tissues

We then constructed a synthetic microbial consortium (BL6) consisting of these six strains and evaluated the effect of BL6 on alleviating *Salmonella* infection in chickens. Given that the first 7 d after hatching represent a critical period for intestinal microbiota colonization in chicks [37], we exploited this “priority effect” to suppress pathogen colonization. Thus, the newly hatched (1-day-old) chicks were used as the model for early intervention. Briefly, chicks received daily oral gavage of BL6 for 4 d, followed by challenge with *S*. Typhimurium for 2 d. Chicks were euthanized at 1 and 7 d post-challenge (Fig. 4A). No mortality was observed in chicks challenged with *S.* Typhimurium during the experimental period. However, challenged chicks exhibited obvious clinical signs of diarrhea compared with the control group. Subsequently, we assessed the effect of early BL6 intervention on intestinal physiology through histological analysis (Fig. 4B and C). In the PC group challenged with *S.* Typhimurium, a notable deepening of the ileal crypts was observed at 7 days of age. Compensatory hyperplasia of crypts is a common mucosal repair response in cases of enteritis. Furthermore, at one and 7 d post-challenge, the BL6 group exhibited a significantly higher villus-crypt ratio than both the CON and PC groups. These results indicated that BL6 has the ability to maintain the gut physiological integrity of *S.* Typhimurium challenged chicks.

### BL6 safeguards liver function and intestinal barrier and alleviates systemic inflammation caused by *Salmonella* infection

**Fig. 5:**
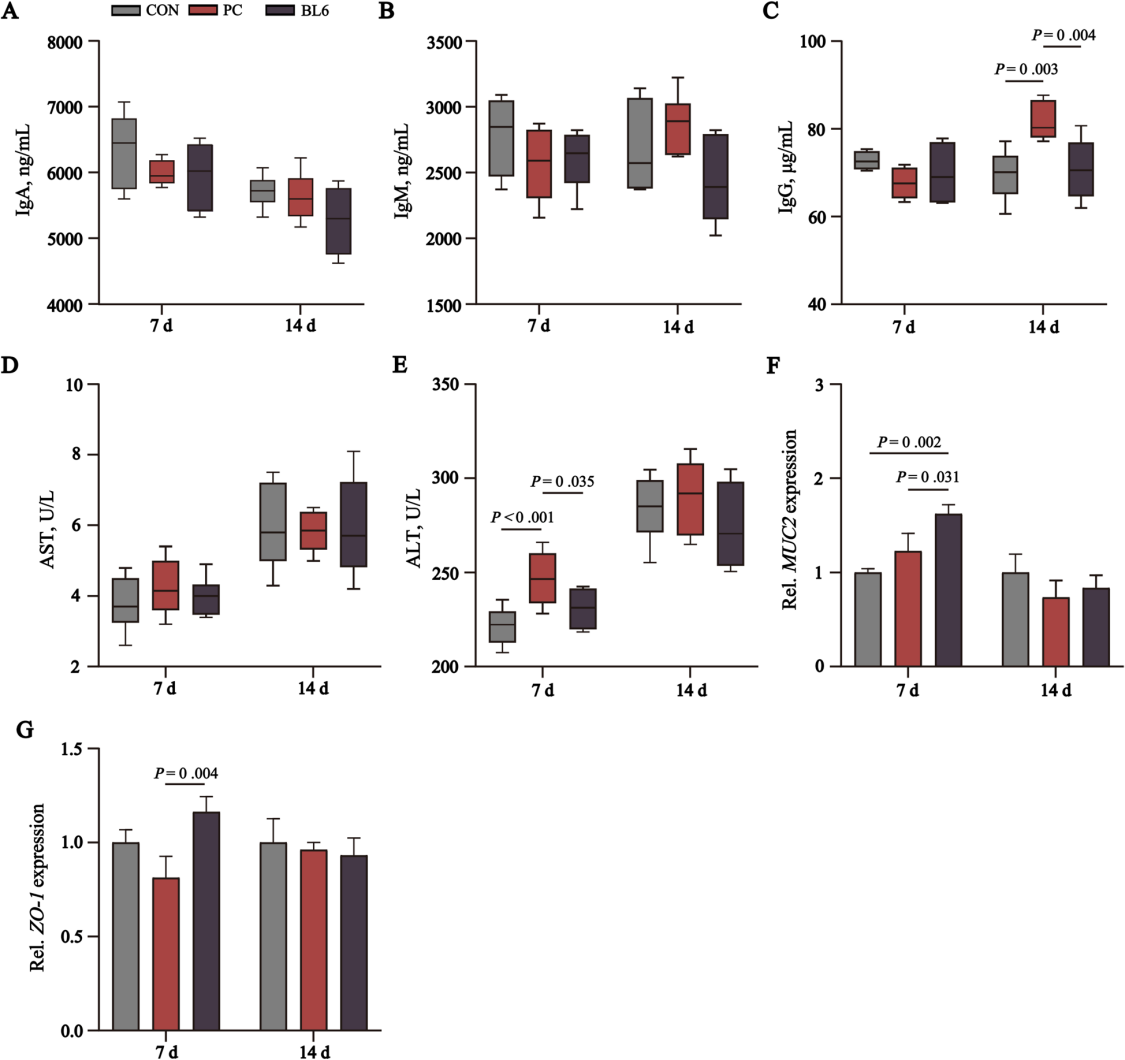
BL6 provides systemic protection by modulating humoral immunity, liver function, and gut barrier during *Salmonella* infection. **A**–**C** Serum IgA, IgM and IgG concentrations at 7 and 14 days of age.** D** and **E** Serum AST and ALT levels.** F** and **G** Relative expression level of *MUC2* and *ZO-1*

We examined the serum antibody responses of chicks using an ELISA kit. The results showed that *Salmonella* challenge did not induce changes in IgA and IgM levels (Fig. 5A and B). However, in 14-day-old chicks, the serum IgG level in the *Salmonella*-challenged group (PC) increased significantly, while that in the BL6 group maintained at a normal level (Fig. 5C). This suggests that *S*. Typhimurium stimulated the humoral immunity of the chicks, but BL6 did not necessitate an excessive immune activation to counteract the challenge. We then measured the AST and ALT concentrations in serum, which are key indicators of liver function. In contrast to the CON group, the PC group showed a notable increase in serum ALT levels at day 1 post-challenge, whereas the BL6 group did not exhibit such a significant elevation (Fig. 5E). These results demonstrated that BL6 could effectively protect the liver from *S*. Typhimurium invasion. Since BL6 demonstrated potential in inhibiting *S.* Typhimurium in both in vitro and in vivo experiments, we investigated whether early intervention with BL6 could enhance the intestinal barrier during *S*. Typhimurium infection. The relative expression levels of genes associated with the intestinal barrier in chick ileum were quantified using RT-qPCR. As expected, the expression of *MUC2* and *ZO-1* genes was significantly upregulated in the BL6 group compared with the PC group at 7 days of age (Fig. 5F and G). Taken together, these findings indicate that BL6 confers multifaceted protection against *Salmonella* infection in chicks, including enhanced intestinal barrier function, hepatic protection, and humoral immunomodulation, all contributing to reduced infection-related damage.

### BL6 restructures the gut microbiota to favor sustained *Lactobacillus* enrichment

**Fig. 6:**
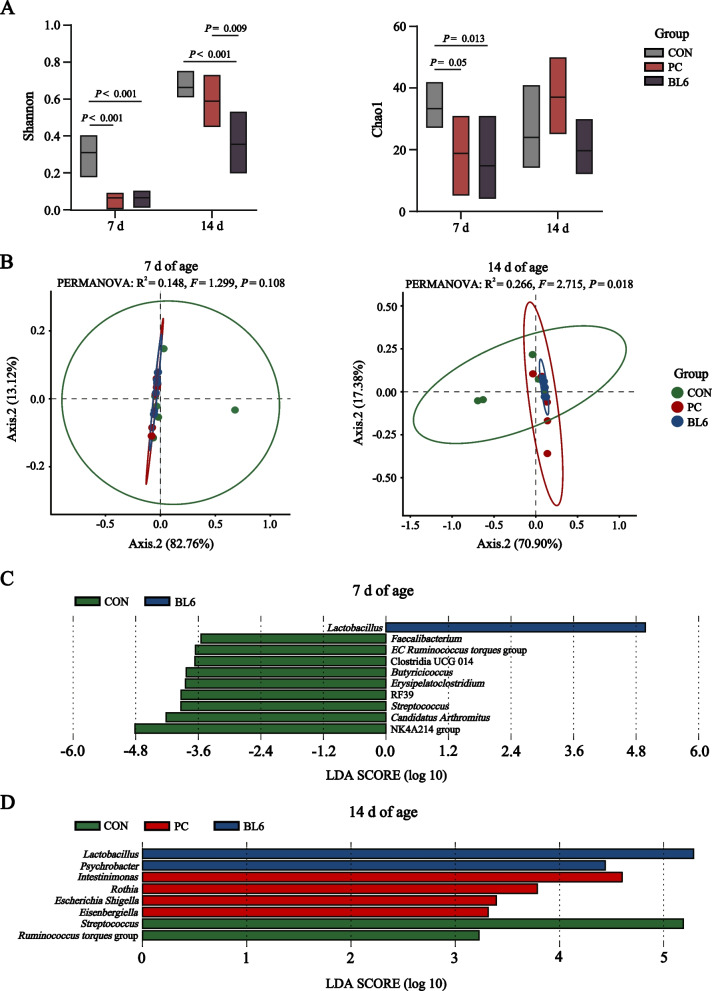
BL6 shapes a *Lactobacillus* promoting gut microbiota. **A** Alpha-diversity indices (Shannon and Chao1) of the ileal microbiota. **B** Beta-diversity visualized by PCoA of ileum communities. **C** and **D** Differentially enriched taxa in the ileal microbiota were identified by LEfSe analysis at 7 (**C**) and 14 days (**D**) of age

We then evaluated the effects of early BL6 intervention on the gut microbiota of chicks through 16S rRNA amplicon sequencing of ileal digesta. At 7 days of age, both the *Salmonella*-challenged PC and BL6 groups exhibited lower Shannon and Chao1 indices than the CON group (Fig. 6A). By 14 days of age, the Chao1 index in both the PC and BL6 groups had returned to normal levels. However, the Shannon index in the BL6 group was significantly lower than that in the CON and PC groups. At this stage, significant differences in community structure were observed among the three groups (Fig. 6B). The PC group maintained a disrupted microbiota structure due to the persistent effect of *Salmonella*, whereas the BL6 group partially restored to a healthy intestinal microbiota structure. These results indicate that early *Salmonella* challenge reduces the diversity of the intestinal microbiota. In contrast, BL6 appears to exert its protective effects through functional pathways independent of restoring diversity. We next characterized the differentially abundant bacterial genera among the three groups using LEfSe analysis. In the BL6 group, *Lactobacillus* began to be enriched in 7-day-old chicks and remained until 14 days of age (Fig. 6C and D). In contrast, in 14-day-old chicks, the PC group showed significant enrichment of pathogenic bacteria (*Escherichia-Shigella*), inflammation-related bacteria (*Rothia*), and bacteria associated with metabolic disorders (*Eisenbergiella*, *Intestinimonas*), indicating an obvious dysbiosis in this group. These results indicate that BL6 can modulate the gut microbiota dysbiosis in chicks caused by *Salmonella* infection, particularly through enriching *Lactobacillus.*

## Discussion

Over the past few decades, extensive investigations into the microbiome have highlighted that changes in the gut microbiota directly affect pathogen colonization in the host [38]. Fecal microbiota transplantation from healthy adults has been applied to inhibit *Clostridioides difficile* infection in humans [6] and to prevent *Salmonella* colonization in chicks [7]. In contrast to the utilization of the entire gut microbiota, in recent years, more trials have been conducted employing precision microbiome reconstitution to prevent pathogen colonization and infection through a single species or defined combination of species [8]. Although chickens are economically significant livestock, the understanding of their gut microbial communities remains considerably less comprehensive compared to that of mice and humans. Certain local chicken breeds, such as Wenchang chicken and Danzhou chicken, may harbor intestinal microbial resources with the potential to combat pathogen infection, owing to their strong environmental adaptability. Based on this premise, we investigated the microbial community structure in adult Wenchang chicken and Danzhou chicken and screened pure cultures with pathogen-exclusion activity from their intestines. Subsequently, we constructed the synthetic consortium BL6 consisting of strains with high anti-*Salmonella* activity and evaluated its role in protecting chicks from *Salmonella* infection. Our study provides new research directions and application avenues for the poultry industry to address the issue of *Salmonella* infections in chickens.

Our results showed that the gut microbial community structure was very distinct between the Wenchang chicken and Danzhou chicken. Given that these two chicken breeds were raised under different dietary and environmental conditions in this study, the observed differences in their gut microbiomes may be attributed to multiple factors, including breed and diet [39]. Previous studies have shown that different chicken breeds exhibit unique gut microbial profiles even when housed in the same facility and fed identical diets [40]. In addition to genetic background, both short-term and long-term dietary patterns are known to shape the composition of the gut microbiota [41–43]. Our results showed that the ileal digesta of Danzhou chicken displayed a higher abundance of *Lactobacillus* (94.73%). In contrast, *Lactobacillus* (34.04%) and *Romboutsia* (30.80%) were the dominant genera in the ileum of Wenchang chicken. *Romboutsia* is closely associated with lipid metabolism [44] and carbohydrate metabolism [45], and is notably enriched in obese individuals, suggesting a potential role in abdominal fat deposition in Wenchang chicken. *Bacteroidetes* and Rikenellaceae RC9 gut group were found to have the highest relative abundance in the cecal digesta of both breeds. These beneficial bacteria are known to contribute to host energy metabolism and intestinal health through the production of short-chain fatty acids (SCFAs) [46]. Further studies are required to elucidate the mechanisms by which these key microbial taxa modulate gut health and the phenotypes of the local chicken breeds.

Given that *Salmonella* infections cause substantial economic losses to the global poultry industry, and based on the concept of “precision microbiome reconstitution”, we postulate that a synthetic microbial consortium assembled by combining multiple anti-*Salmonella* bacteria may offer enhanced *Salmonella*-inhibiting capacity. From our cultured collection of 273 isolates, we screened 6 strains with the strongest in vitro inhibitory activity against *S.* Typhimurium as candidates for BL6 construction. Among these six strains, four were LAB and two were *Bacillus*. Oral administration of LAB has frequently been shown to increase the levels of SCFAs in feces, consequently inhibiting *Salmonella* through acidity-related mechanisms [47]. LAB are also known to produce bacteriocins, which are peptides with antibacterial activity [48]. For instance, the BCN OR7 bacteriocin produced by *Lactobacillus salivarius* protects chickens against *Campylobacter jejuni* infection [49]. In addition, certain LAB can protect the gut against pathogenic invasion through hydrogen peroxide production, potentially involving hydroxyl radical-induced DNA damage [50]. As for *Bacillus* bacteria, numerous studies have demonstrated their excellent anti-pathogen properties. Certain *Bacillus* bacteria play a crucial role in maintaining the anti-infectious functions of the gastrointestinal tract by inhibiting the adhesion of pathogens to the mucosal surface. In addition to this indirect effect, they also secrete secondary metabolites, such as fengycin, which directly inhibit the proliferation of pathogens [51]. In this study, we also predicted multiple genes encoding anti-*Salmonella* antimicrobial peptides and secondary metabolite gene clusters involved in the biosynthesis of bacilysin, fengycin, and bacillaene in the genomes of the *Bacillus* strains. Furthermore, bacillibactin encoding genes were found in the genomes of both *Bacillus* strains (Table S7). As a type of siderophore, bacillibactin aids bacteria in iron acquisition by chelating environmental iron ions [52], thereby enhancing their competitive advantage in nutrient competition. During infection, *S.* Typhimurium actively takes up substantial amounts of iron from the inflamed gut [53]. The presence of *Bacillus* bacteria secreting bacillibactin in the gut can potentially sequester iron. This sequestration reduces the availability of iron for *S*. Typhimurium, thereby disrupting its normal physiological functions and limiting its ability to establish a successful infection. *Escherichia coli* Nissle 1917, utilizing similar iron-assimilation pathways, is able to outcompete *S*. Typhimurium and reduce its colonization in mouse models of acute colitis and chronic persistent infection [54, 55].

Previous studies have reported negative effects of *Salmonella* challenge on mortality and growth performance [56, 57]. The mortality rate of *Salmonella*-infected chicks can reach up to 50% in the absence of treatment [57]. The mortality of challenged chicks may be related to the challenge dose [58], exposure age [59], and virulence [60] of the *Salmonella* strain used. In our study, chicks inoculated with *Salmonella* exhibited clinical signs including diarrhea, reduced activity, isolation, or lethargy, but no mortality was observed. This is consistent with our experimental model, which induced intestinal infection manifesting as diarrhea rather than lethal systemic disease. In the present study, *Salmonella* challenge markedly reduced the growth performance of chicks, while BL6 administration tended to alleviate growth depression (Fig. S5). However, the trial period was limited to 14 d, and further studies with a longer duration are needed to confirm the long-term effects of BL6. BL6 showed positive effects for challenged chicks, indicating its anti-infective efficacy against *Salmonella*. BL6 was subsequently tested using a 1-day-old chick model to evaluate its potential to exclude *Salmonella* from the chick gut and promote gut health. Our results clearly showed that BL6 reduced the tissue damage and enhanced the intestinal barrier function of chicks (Figs. 4B and C, 5F and G). ZO-1 maintains the integrity of tight junctions in the intestinal epithelial cells, while mucin MUC2 secreted by goblet cells forms a protective layer covering intestinal epithelial cells, thereby preventing pathogen invasion [61, 62]. Promising results were observed regarding the enhancement of intestinal barrier function, though this effect was transient (detected at 7 but not at 14 days of age). Despite the lack of a long-lasting effect, the mRNA expression levels in the BL6 group at the earlier infection time point may still confer enhanced resistance during a phase when chicks are particularly susceptible to pathogens. In poultry, the effects of enteric pathogens are not always overt, even when chickens do not exhibit clinical signs, these pathogens may still negatively impact the host. It has been shown that impairment of the intestinal barrier can enhance pathogen access to underlying tissues and activate host immune compartments, which in turn impairs nutrient absorption while increasing the availability of growth substrates required for pathogen proliferation [63, 64]. In our study, the improved integrity of intestinal villi and intestinal barrier may explain how BL6 enhances intestinal resistance in chicks during *Salmonella* challenge. The positive role of BL6 in reducing *Salmonella* infection can also be reflected by the immune responses in our study. Previous research has shown that injection of heat-inactivated *Salmonella* increases IgG levels [65]. Similarly, we observed a rise in serum IgG following oral administration of *Salmonella*. In the challenged group, *Salmonella* proliferation triggers the secretion of IgG, whereas in the BL6 group, the stimulatory signal for IgG production was blocked.

Our results further demonstrated that BL6 exerted a regulatory effect on the gut microbiota. 16S rRNA amplicon sequencing of the gut microbial community structure revealed that during the short-term challenge with *S. Typhimurium* (1 d post-challenge), the microbial richness and diversity in both challenged groups decreased, whereas the BL6 group had already initiated the proliferation of beneficial *Lactobacillus* (Fig. 6A and C). In the longer term (7 d post-challenge), the BL6 group remained a lower Shannon index after exposure to *S.* Typhimurium (Fig. 6A). Nevertheless, the BL6 group restructured the microbiota into a beneficial bacterium-dominated equilibrium by promoting the proliferation of *Lactobacillus* and facilitating their dominance, while the PC group remained in a pathogen-dominated state (Fig. 6C). Given that BL6 contained four LAB strains (*L. johnsonii* CMLH106, *L. crispatus* CMLH109, *L. gallinarum* CMLH110, *L. salivarius* CMLH112), the increase in *Lactobacillus* in the BL6 chicken group is likely attributable to our intervention. Although two *Bacillus* bacteria (*Bacillus* sp. CMLH124 and *B. velezensis* CMLH125) were also incorporated into BL6, the genus of *Bacillus* was not found to be increased in the BL6 group. A future strain-level analysis of the gut microbiota will aid in determining which members in BL6 were successfully colonized in the chicken gut. Furthermore, although we initially intended to include more *Salmonella*-inhibiting strains in BL6 to enhance the pathogen-eliminating effect, whether a reduced number of strains or even only *Lactobacillus* strains can achieve comparable results to the current six-member BL6 warrants further investigation.

## Conclusion

In this study, we characterized the gut microbial community structures of two Chinese local chicken breeds. From the culturable bacteria of these chickens, we constructed a consortium named BL6, comprising six strains with high anti-*Salmonella* activity. BL6 effectively inhibits the colonization of *Salmonella* and protects chicks against *Salmonella*-induced pathological changes. In addition, BL6 exerts a regulatory effect on the chicken gut microbiota by significantly enriching the abundance of *Lactobacillus*. Overall, this study provides compelling evidence that a specific microbial community, assembled using strains from local chicken breeds, has the potential to eliminate *Salmonella* infection in chicks, offering a practical strategy for improving the gut health of chickens and decreasing the prevalence of *Salmonella* in the poultry industry.

## Supplementary Information

Additional file 1: Table S1. Composition of different media. Table S2. Taxonomic affiliation of cultures (EzBiocloud database). Table S3. Candidates for novel species (NCBI database). Table S4. Inhibition zone diameters of isolates against three pathogenic bacteria. Table S5. Number of predicted genes encoding CAZymes in the six strains. Table S6. Predicted antimicrobial peptides in six strains. Table S7. Predicted secondary metabolite biosynthetic gene clusters in CMLH124 and CMLH125. Table S8. Composition and nutrient level of experimental diets. Table S9. Target gene primer sequence.

Additional file 2: Fig. S1. Characteristics of phylum composition in the gut microbiota of ileum and cecum in Danzhou chicken and Wenchang chicken. Fig. S2. Species features of circular genome of six strains. Fig. S3. KEGG pathway annotation of six strains. Fig. S4. COG pathway annotation of six strains. Fig. S5. The effects of BL6 on growth performance of *Salmonella* challenge chicks.

## Data Availability

The datasets used and analyzed during the current study are available from the corresponding authors on reasonable request.

## Abbreviations

ALT: Alanine aminotransaminase
AST: Aspartate aminotransaminase
CAZyme: Carbohydrate-active enzymes
COG: Clusters of orthologous groups of proteins
ELISA: Enzyme-linked immunosorbent assay
GAM: Gifu anaerobic medium
GH: Glycoside hydrolase
GT: Glycosyl transferase
IgA: Immunoglobulin A
IgG: Immunoglobulin G
IgM: Immunoglobulin M
KEGG: Kyoto Encyclopedia of Genes and Genomes
LAB: Lactic acid bacteria
LB: Luria–Bertani
LDA: Linear discriminant analysis
LEfSe: Linear discriminant analysis effect size
MRS: De Man, Rogosa and Sharpe
MUC2: Mucin 2
PBS: Phosphate Buffer Saline
PCoA: Principal coordinate analysis
RT-qPCR: Reverse transcription-quantitative real-time polymerase chain reaction
SEM: Standard error of the mean
ZO-1: Zonula occludens 1
