# Supplementary material for: A microbial consortium constructed with gut microbes of Chinese native chicken breeds protects chicks against Salmonella infection
Source: J Anim Sci Biotechnol. 2026 Jun 26;17:130. doi: 10.1186/s40104-026-01447-2 (PMC13307419; doi:10.1186/s40104-026-01447-2)
Supplement: Supplementary file 2 — Additional file 2: Fig. S1. Characteristics of phylum composition in the gut microbiota of ileum and cecum in Danzhou chicken and Wenchang chicken. Fig. S2. Species features of circular genome of six strains. Fig. S3. KEGG pathway annotation of six strains. Fig. S4. COG pathway annotation of six strains. Fig. S5. The effects of BL6 on growth performance of Salmonella challenge chicks. [file 40104_2026_1447_MOESM2_ESM.docx]

**
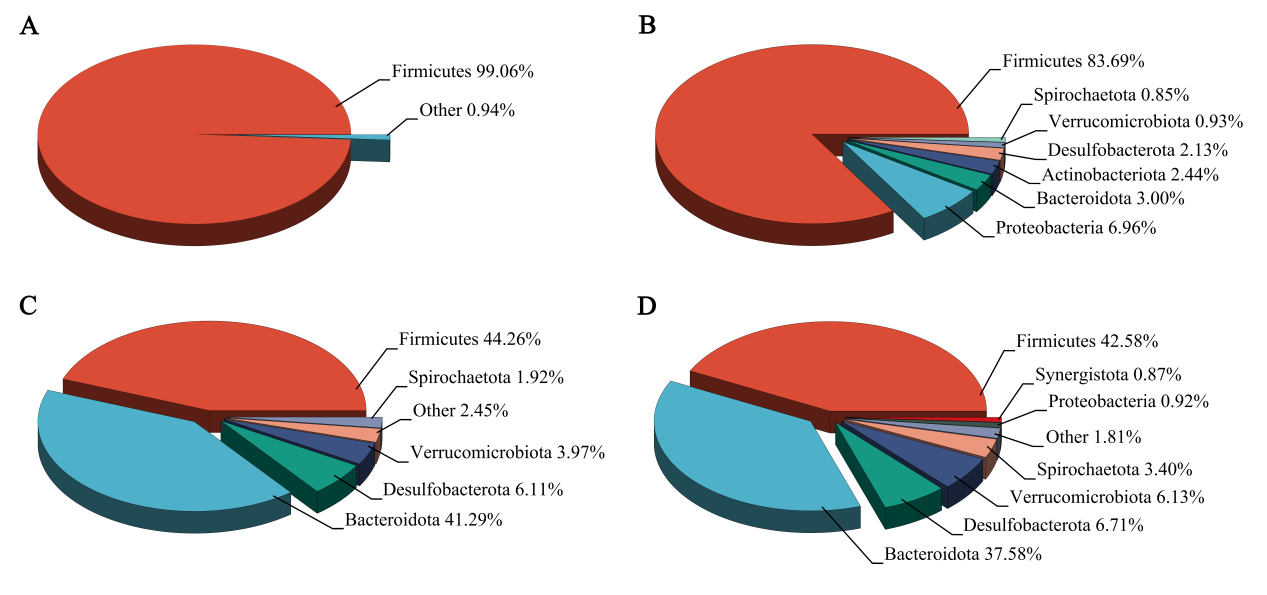
**

Fig. S1. Characteristics of phylum composition in the gut microbiota of ileum and cecum in Danzhou chicken and Wenchang chicken. **A** and **B** Pie chart of the relative abundance of ileal microbial phyla in Danzhou chicken (A) and Wenchang chicken (B). **C** and **D** Pie chart of the relative abundance of cecal microbial phyla in Danzhou chicken (C) and Wenchang chicken (D). **
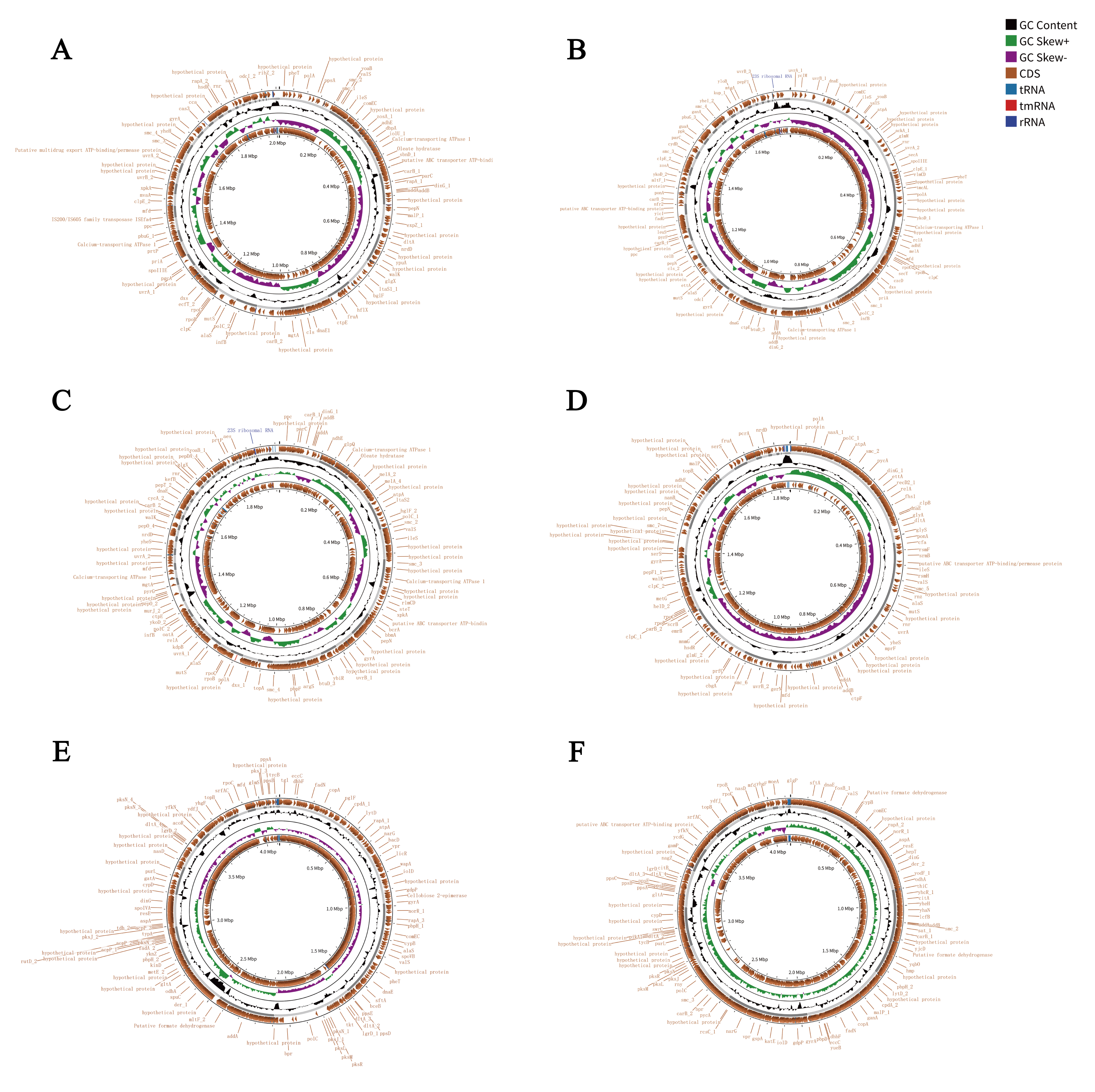
**

Fig. S2. Species features of circular genome of six strains. **A** *L. johnsonii* CMLH106. **B** *L. crispatus* CMLH109. **C** *L. gallinarumd* CMLH110. **D** *L. salivarius* CMLH112. **E** *Bacillus* sp. CMLH124. **F** *B. velezensis* CMLH125. The brown circle represents the coding regions (CDS) and non-coding RNA regions (including rRNA, tRNA, and tmRNA) of the strains. The outermost circle denotes CDS located on the antisense strand of the DNA molecule, while the innermost circle denotes CDS located on the sense strand. The green and purple circles respectively represent the G+C skew, and the black circle represents the G+C content.

**
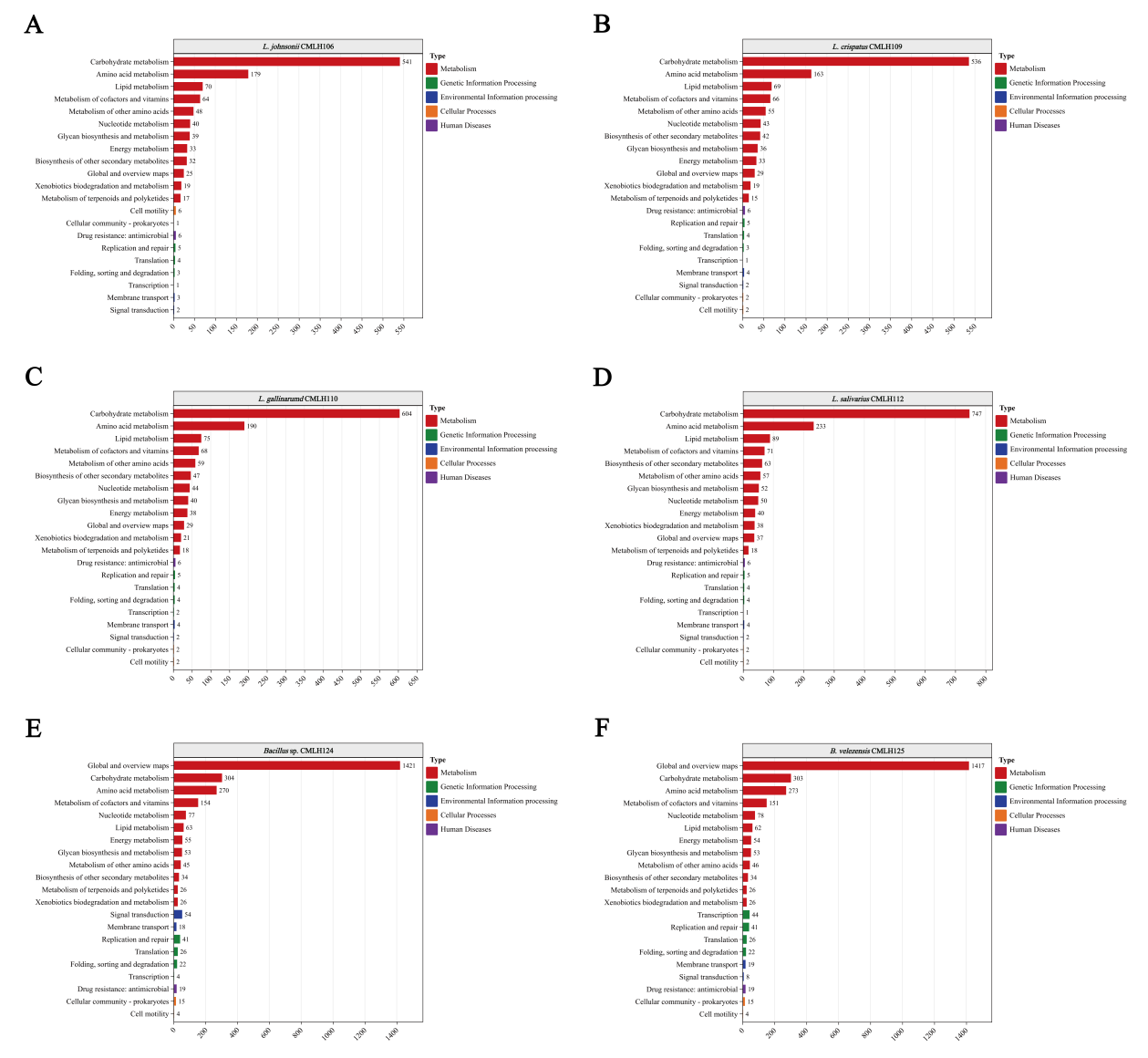
**

Fig. S3. KEGG pathway annotation of six strains. **A** *L. johnsonii* CMLH106. **B** *L. crispatus* CMLH109. **C** *L. gallinarumd* CMLH110. **D** *L. salivarius* CMLH112. **E** *Bacillus* sp. CMLH124. **F** *B. velezensis* CMLH125. Among the six strains, a total of 1138, 1135, 1264, 1525, 2731, and 2721 genes from their genomes were respectively annotated to 21 KEGG pathways, which belong to five major functional categories: metabolism, cellular processes, human diseases, genetic information processing, and environmental information processing. Notably, over 92% of these genes are involved in metabolic processes. At the sub-classification level of metabolic pathways, carbohydrate metabolism and amino acid metabolism are the main annotated pathways for CMLH106, CMLH109, CMLH110, and CMLH112. Meanwhile, the primary annotated pathways for CMLH124 and CMLH125 are global and overview maps and carbohydrate metabolism.

**
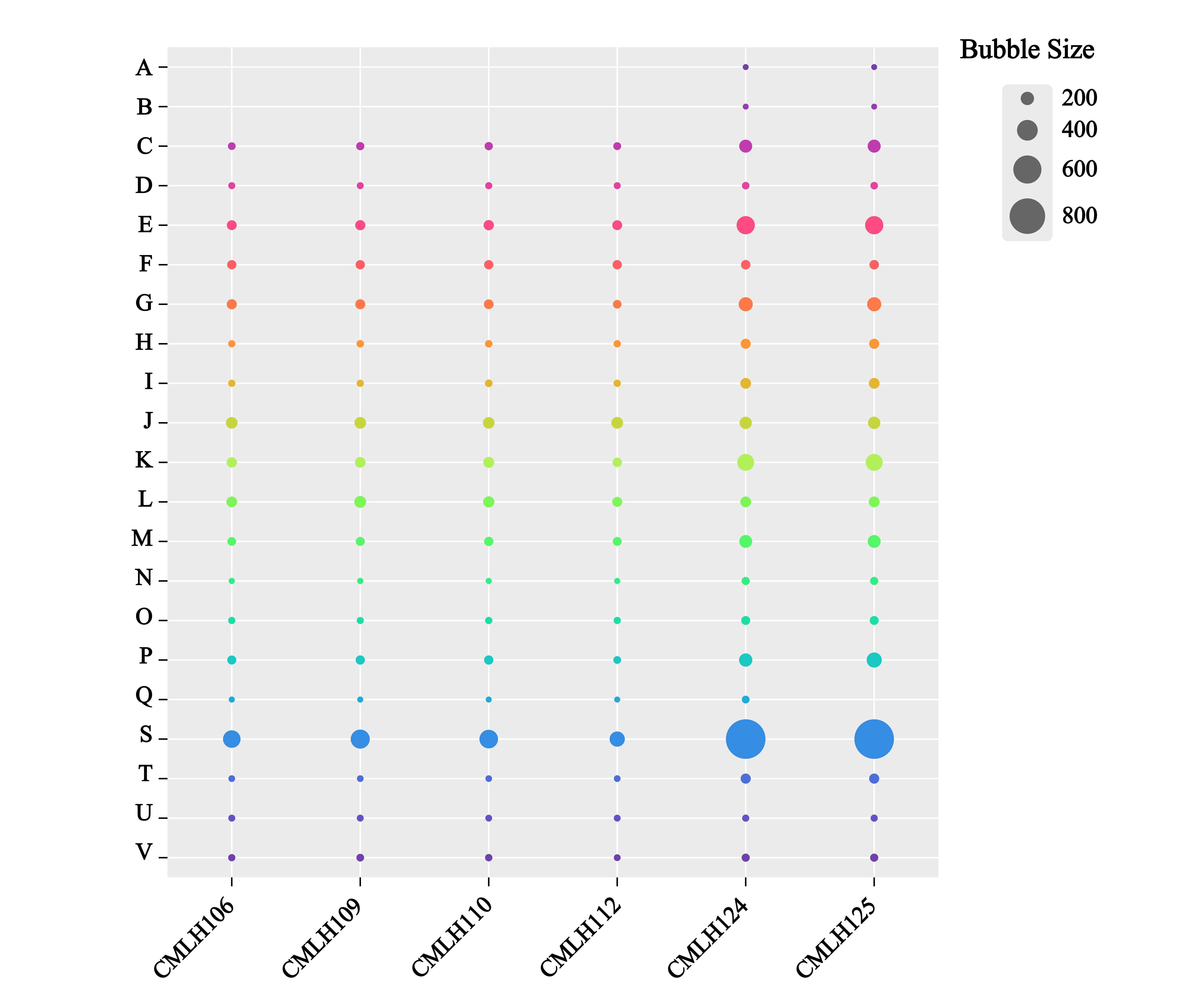
**

Fig. S4. COG pathway annotation of six strains. A, RNA processing and modification. B, Chromatin structure and dynamics. C, Energy production and conversion. D, Cell cycle control, cell division, chromosome partitioning. E, Amino acid transport and metabolism. F, Nucleotide transport and metabolism. G, Carbohydrate transport and metabolism. H, Coenzyme transport and metabolism. I, Lipid transport and metabolism. J, Translation, ribosomal structure and biogenesis. K, Transcription. L, Replication, recombination and repair. M, Cell wall/membrane/envelope biogenesis. N, Cell motility. O, Posttranslational modification, protein turnover, chaperones. P, Inorganic ion transport and metabolism. Q, Secondary metabolites biosynthesis, transport and catabolism. R, General function prediction only. S, Function unknown. T, Signal transduction. U, Intracellular trafficking, secretion, and vesicular transport. V, Defense mechanisms. Among the six strains, 1464, 1595, 1578, 1285, 3418 and 3419 genes were respectively annotated to 21 COG functional categories. Excluding genes with unknown functions, the most abundant functional categories in CMLH106, CMLH109, CMLH110, and CMLH112 were translation, ribosomal structure and biogenesis, replication, recombination and repair, transcription, carbohydrate transport and metabolism, amino acid transport and metabolism. The most abundant categories in CMLH124 and CMLH125 were amino acid transport and metabolism, transcription, carbohydrate transport and metabolism, and inorganic ion transport and metabolism.


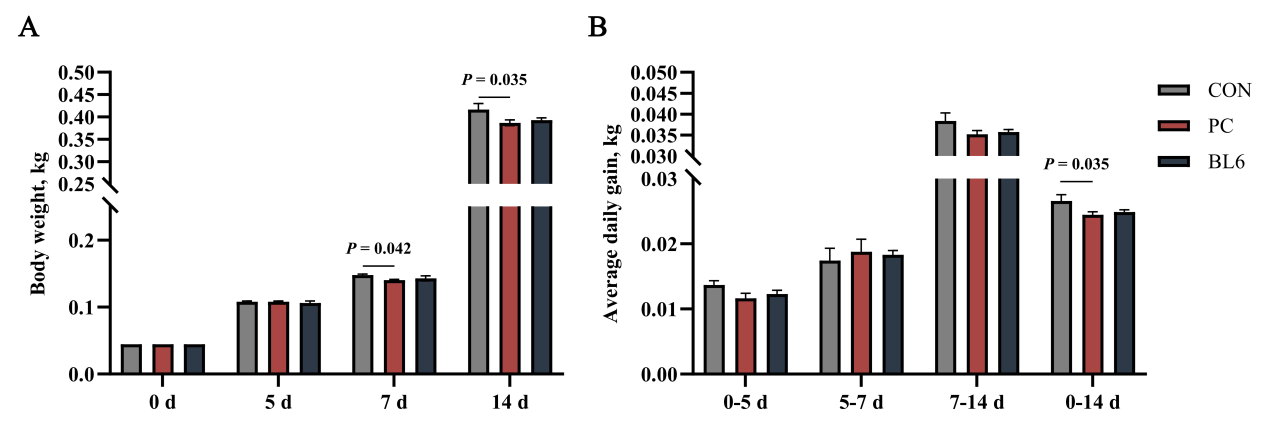


Fig. S5. The effects of BL6 on growth performance of *Salmonella* challenge chicks. **A** Body weight of chicks at different time points. **B** Average daily gain of chicks at different stages.
